# Cocaine in Dental Practice

**Published:** 1885-04

**Authors:** 


### COCAINE IN DENTAL PRACTICE.

Probably quite enough has been said upon the subject of Cocaine to enable the profession to comprehend its proper place in the dental pharmacopœia. Sufficient of testimony has been given, and a fair verdict upon its merits may be pronounced without much risk. It is not all that the profession has been looking and hoping for. It is not entirely reliable as an obtundent of dentinal tissues. It *is* a local anæsthetic, probably the most potent in its effects upon soft tissues of anything yet discovered. Its results are, however, principally confined to such tissues as have a distinct nervous supply. The manner in which Cocaine, a drug that was announced as a local anæsthetic, was received by the dental profession, and the sanguine expectations and even confident assertions of what would be and were its results when applied to non-vascular and nerveless tissues, is rather a severe comment upon the heedless and unscientific way in which we too often use drugs.

All true anæsthetics paralyze nerve tissue, commencing at the periphery and proceeding toward the nerve centers. Their specific effect is upon the nerves themselves, and they are brought in contact with them by being carried through the arterial supply, and hence their first impression is upon the terminal filaments. Local anæsthetics, on the contrary, are not distributed through the general circulation, but are placed in direct contact with the peripheral ends of the nerves at once, and manifest their peculiar properties only upon the nerves which are thus brought within their immediate influence. Cocaine is such a local anæsthetic, and when tissues having a nervous supply are submitted to its action, either by hypodermic injection or through absorption from topical application, its regular characteristic effects are manifested.

The great desideratum in dentistry is a drug that shall obtund sensitive dentine, a tissue that is without nervous supply. In just what manner sensation is conveyed through the protoplasmic and nerveless dentinal fibrils, has not yet been ascertained. When this problem has been solved, we shall be much nearer the discovery of a reliable obtunder of sensitive dentine. In the meantime, we are grasping at straws in our search for a grand specific that shall combine quite contradictory qualities. Without the knowledge that should enable us scientifically to apply a proper remedy, we are empirically trying the most diverse drugs. More than one formula has been confidently offered, that was composed of drugs that were glaring "incompatibles." We are in the condition of physicians who are blindly prescribing without having been able to make a diagnosis. The first thing to be done is to determine the exact nature of the dentinal filaments, and their precise connection with the nerves themselves. Much has been done during the past few years, but more remains to be accomplished. When we are intimately acquainted with the tissues with which we have to deal, we may hope to intelligently apply a remedy for any pathological condition.

Cocaine has no more effect upon nerveless tissues than many other things that might be named. For obtunding dentine it is of no more use than are some remedies that are not local anæsthetics at all. It is the universal experience with such obtunders as have attained any degree of popularity at all, that in some cases they are perfectly effective, and in others as complete failures. This seems to be the fact with Cocaine. As a local anæsthetic, the results of its use upon soft tissues are amazing. In certain teeth of a loose histological structure, teeth in which the living portion has a high organization while the inorganic is deficient and poorly crystallized, the application of Cocaine has produced marked results. But in such cases alcohol, and ether, and chloride of zinc, and preparations of morphia, and chloral-hydrate, and many others, have been known to work wonders. Yet none of these are unfailingly and in all cases effective, and Cocaine seems to possess comparatively little advantage over some of them in this respect.

As a local anæsthetic for soft tissues, it will always occupy a distinguished place, but as an obtundent of sensitive dentine it cannot be unfailingly relied upon.

